# Long-Tailed Unconventional Class I Myosins in Health and Disease

**DOI:** 10.3390/ijms21072555

**Published:** 2020-04-07

**Authors:** A. Navinés-Ferrer, M. Martín

**Affiliations:** 1 Biochemistry and Molecular Biology Unit, Biomedicine Department, Faculty of Medicine, University of Barcelona, 08036 Barcelona, Spain; 2 Laboratory of Clinic and Experimental Respiratory Immunoallergy, IDIBAPS, 08036 Barcelona, Spain; 3 ARADyAL research network, Carlos III Health Institute, 28029 Madrid, Spain

**Keywords:** unconventional myosins, integrins, adaptor molecules, immune cells, cell adhesion, migration, phagocytosis, host defense, cancer

## Abstract

Long-tailed unconventional class I myosin, Myosin 1E (MYO1E) and Myosin 1F (MYO1F) are motor proteins that use chemical energy from the hydrolysis of adenosine triphosphate (ATP) to produce mechanical work along the actin cytoskeleton. On the basis of their motor properties and structural features, myosins perform a variety of essential roles in physiological processes such as endocytosis, exocytosis, cell adhesion, and migration. The long tailed unconventional class I myosins are characterized by having a conserved motor head domain, which binds actin and hydrolyzes ATP, followed by a short neck with an isoleucine-glutamine (IQ) motif, which binds calmodulin and is sensitive to calcium, and a tail that contains a pleckstrin homology domain (PH), a tail homology 1 domain (TH1), wherein these domains allow membrane binding, a tail homology 2 domain (TH2), an ATP-insensitive actin-binding site domain, and a single Src homology 3 domain (SH3) susceptible to binding proline rich regions in other proteins. Therefore, these motor proteins are able to bind actin, plasma membrane, and other molecules (adaptor, kinases, membrane proteins) that contribute to their function, ranging from increasing membrane tension to molecular trafficking and cellular adhesion. MYO1E and MYO1F function in host self-defense, with a better defined role in innate immunity in cell migration and phagocytosis. Impairments of their function have been identified in patients suffering pathologies ranging from tumoral processes to kidney diseases. In this review, we summarize our current knowledge of specific features and functions of MYO1E and MYO1F in various tissues, as well as their involvement in disease.

## 1. Unconventional Myosins: General Introduction

The myosin protein superfamily found in eukaryotic cells comprises at least 18 classes [1]. Myosins are motor proteins that bind actin filaments (F-actin) in an adenosine triphosphate (ATP)-regulated manner. Binding of F-actin promotes ATP hydrolysis by myosin that, in turn, can power movement of the actin filament or movement of the myosin along the filaments. Structurally, these proteins have an N-terminal “head”, a middle “neck” region, and a C-terminal “tail” [1].

The N-terminal head is responsible for motor activity, containing a nucleotide binding pocket, where ATP hydrolysis occurs, and has an actin binding site. Most myosins contain between one and seven isoleucine-glutamine (IQ) motifs characterized by the IQXXXRGXXXR sequence [2] in the neck region, and these follow the conserved motor domain sequences directly. Often, calmodulin (CaM) or CaM-related proteins are associated with stabilizing the α-helical conformation adopted by these IQ motifs [3,4]. In fact, the IQ motifs and bound light chains form a rigid structure that serves as a mechanical lever [5]. The number of IQ motifs determines the length of the lever arm and, therefore, the step-size and the force of the myosin motor [6]. The C-terminal tail is the region that diverges most among myosins, having a role in dimerization and being responsible for binding to a diverse array of partners, targeting each myosin to its particular subcellular location and regulatory function [7].

An evolutionary hypothesis supports that class I and II myosins evolved first among all myosins. Class II myosins were the first to be discovered and are considered “conventional”, with all other classes considered “unconventional”. The conventional myosins form large bipolar filaments via tail-directed homo-oligomerization, whereas the unconventional myosins do not form filaments, although some can dimerize, and their tails bind directly to the membrane or other proteins [8]. Unconventional myosins may function to move membranous organelles along actin filaments. Using the “highways and local roads” analogy, microtubules serve as long range highways for organelle transport powered by the motor proteins kinesin or dyneins (opposite direction), whereas F-actin serves as short range roads managed by unconventional myosins [9]. In addition, these myosins can play roles other than cellular trafficking, such as exocytosis, endocytosis, signal transduction, cell adhesion, and cell migration. To perform these diverse mechanical tasks, a large number of specialized myosin isoforms have evolved, which are adapted for high-speed force generation, processive movement over long distances, tension sensing, and linking signal pathways to motor activity [10].

A striking feature of myosin motors is the great variability in the length of the neck region formed by the α-helix of IQ motifs, which are stabilized by binding light chains of the calmodulin family. Variations in these light chain motifs and in the length and stiffness of the myosin appear to affect the mechanical properties of the myosin motor [10] and give rise to the different classes of unconventional myosins [11].

In this review, we will focus on class I unconventional myosins, particularly regarding our current knowledge of how long-tailed forms function and are involved in disease.

## 2. Class I Unconventional Myosins

Class I myosins are the largest group of unconventional myosins and are evolutionary ancient, existing in a wide range of species from yeast to vertebrates [12]. Type I myosin functions as a monomer, with one end connecting to lipids in the plasma membrane and the other connecting to cortical actin (i.e., the network of actin filaments underneath the plasma membrane). When myosin type I connects to cortical actin and hydrolyzes ATP, it tucks in and moves along the actin filament (to the plus end) and creates tension but reinforces the connection between the plasma membrane and cortical actin, enabling the cell to support the plasma membrane [13]. 

**Figure 1:**
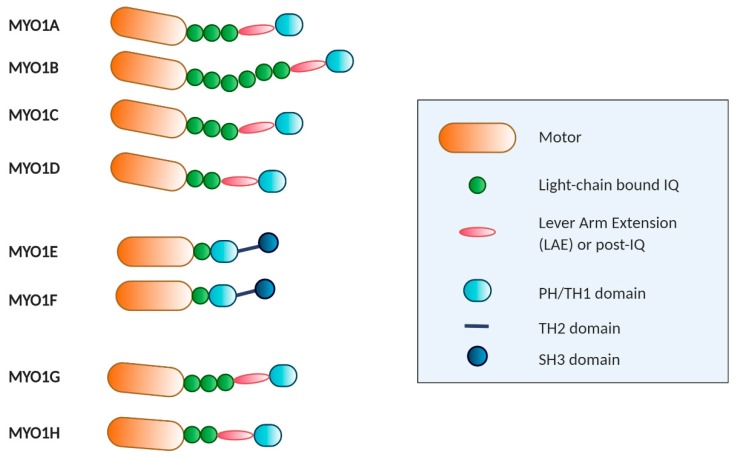
Schematic representation and structure of human unconventional type 1 myosins. All myosins comprise conserved motor heads (orange), different numbers of isoleucine-glutamine (IQ) motifs (green), a helical region (pink), IQ and helical extension comprising the neck region, and distinct tails with various functional domains. Long-tailed myosins, such as MYO1E and MYO1F, also have an Src homology 3 domain (SH3) domain for protein–protein interaction.

Mice and humans have a total of eight class I heavy-chain myosin genes, of which six encode short-tailed forms (Myosin 1A, -B, -C, -D, -G, and -H) and two encode long-tailed forms (MYO1E and MYO1F) [14]. MYO1B and MYO1E are expressed in most cells, but MYO1A and MYO1F are more restricted to the intestines and hematopoietic cells, respectively [15]. Myosin class I associates with between one and six calmodulins, and all have either a pleckstrin homology (PH) domain or a basic tail homology 1 domain (TH1) in their tail that are thought to affect membrane interaction [16]. The PH domain bears the KXn(K/R)XR (where K is lysine, R arginine, X any amino acid) canonical sequence for binding to phosphoinositides and is located between the β1 and β2 sheets within the TH1 domain [17]. TH1 is basic in charge and may effect interactions with negatively charged phospholipids. The lipid specificities and function in membrane interaction are highly dependent on the isoform [11]. The long-tailed forms also contain a proline-rich tail homology 2 domain (TH2) domain; this domain contains an ATP-insensitive actin binding site [18] and a large number of basic amino acids suitable for binding membrane phospholipids [19].The C-terminal end of the tail has a single Src homology 3 (SH3) domain, which binds to proline rich regions, particularly proteins carrying the PXXP (where P is a proline and X any amino acid) motif [20] (Figure 1). 

## 3. The Long-Tailed Unconventional Class I Myosins

MYO1E and MYO1F are long-tailed class I myosins that have similar structures and patterns of tissue expression. Although their similarities reveal some convergences between the two myosins, they have important differences that result in discrepancies in cellular function and their role in diseases. Both MYO1E and MYO1F are expressed mainly in immune system cells. MYO1E has a wider expression pattern than MYO1F, and is highly present in the spleen, mesenteric lymph nodes, and lung, as well as to a lesser extent in the intestines and skin. By contrast, MYO1F is mostly expressed in the spleen, mesenteric lymph nodes, thymus, and lungs [15]. Lymphoid tissues, natural killer cells, macrophages, and dendritic cells express considerable levels of both MYO1E and MYO1F, with selective expression reported in B cells and neutrophils, respectively [15]. Lately, MYO1F has also been reported as being expressed in mast cells [21].

### 3.1. MYO1E and MYO1F in Neutrophils

Neutrophils play an important role in innate immunity, and their extravasation in response to an infection or injury is the factor that contributes fastest to the elimination of a pathogen and subsequent wound healing [22,23]. Neutrophil recruitment to the site of inflammation must be carefully regulated because deficient or excessive levels can have severe pathological consequences. As reported recently, neutrophils can contribute to tissue injury by amplifying the inflammatory response and direct release of toxic effectors and assist in the development of many noninfectious diseases, such as lung injury, autoimmune diseases, and cancer [24]. Neutrophil extravasation implies, among other things, proper adhesion to the vascular endothelium and migration to the infected tissue, which depends on actin remodeling and the regulated action of myosins [25,26]. Motile neutrophils exhibit a polarized morphology characterized by the formation of leading edge pseudopods and a highly contractile cell rear known as the uropod [23]. 

Although MYO1E is barely expressed in neutrophils, it has been shown to be required for their efficient extravasation [27]. Recently, MYO1E-deficient neutrophils were shown to have diminished arrest, spreading, uropod formation, and chemotaxis due to defective actin polymerization and integrin activation. Indeed, β2 integrin-mediated rolling and adhesive interactions are affected in MYO1E knock out neutrophils. This phenotype resulted in increased rolling velocity, decreased firm adhesion, aberrant crawling, and strongly reduced transmigration. Thus, MYO1E appears to regulate the adhesive interactions of neutrophils with the vascular endothelium needed for neutrophil extravasation, reducing both 2D and 3D migration [27].

MYO1F is highly expressed in neutrophils, where it plays an essential role in their migration. Neutrophils from MYO1F-deficient mice showed stronger adhesion to integrin ligands, including intercellular adhesion molecule-1 and fibronectin, and most of this adhesion was mediated by β2 integrin. Indeed, MYO1F-deficient neutrophils exhibited high levels of cell-surface β2 integrin [15]. Given that regulated integrin-mediated adhesion to the vascular endothelium is critical to neutrophil migration to infected tissue, MYO1F-deficient mice unsurprisingly presented higher mortality when exposed to infection by *Listeria monocytogenes*. Similarly, effects on defective neutrophil migration have been found in SH3-binding protein 2 (3BP2)-knockout mice, which also resulted in a higher mortality to *Listeria* infection [28]. Interestingly, the adaptor protein 3BP2 has been reported to be a ligand of MYO1F [21]. However, the mechanistic details of how these binding partners regulate neutrophil migration remains to be elucidated.

In the analysis of neutrophil migration in 3D experiments, transmigration and migration in collagen networks showed that neutrophil extravasation into the tissue was also severely compromised in MYO1F-deficient mice due to a defective dynamic deformation of the nucleus [29]. For successful cell migration in these contexts, the nucleus must undergo defined changes in position and shape that are dependent on cytoskeletal dynamics and the mechanical linkage between actin filaments and the nuclear membrane. MYO1F was found to be enriched at the rear and the front ends of the elongated nucleus during the initiation and deformation phases, and it was probably involved in pushing and/or pulling the nucleus through the constriction sites, transmitting force from the cytoskeleton to the inside of the nucleus [29]. Together, these results support the contention that MYO1F is key to host defenses by facilitating neutrophil migration to the site of inflammation.

The impaired neutrophil migration observed in MYO1E- and MYO1F-deficient mice have distinct molecular foundations. In the case of MYO1F, its absence did not lead to reduced neutrophil rolling or adhesion on endothelial cells, a phenomenon that was described in MYO1E-deficient neutrophils [27]. MYO1F-mediated neutrophil migration has been reported to be critical to acute neuroinflammation in ischemic stroke, directly affecting outcomes. During the acute phase of a stroke, neutrophils from the peripheral blood are the first to arrive in the ischemic brain, which then attracts other immune cells that exacerbate neuroinflammation in the ischemic tissue [30]. Although further research on dissecting the ligand partners and mechanisms will be important to unraveling the causes of the functional differences between MYO1E and MYO1F, data currently points to long-tailed class I myosins having a key role in neutrophil function.

### 3.2. MYO1E and MYO1F in Macrophages

Phagocytosis of invading pathogens and/or cellular debris are processes carried out mainly by macrophages in the different tissues. These events needed for host defense, tissue remodeling, and repair require significant changes in phagocyte morphology that accounts for the coordinated participation of a plethora of molecules involved in adhesion, membrane arrangements, and actin cytoskeleton dynamics [31].

The sensing of infectious danger by macrophages through the ligation of toll-like receptors (TLR) triggers fast and robust cytoskeletal changes, including an integrin-mediated spreading response that is dependent on actin polymerization [32,33]. MYO1E, along with its closely related family member MYO1F, are strongly serine phosphorylated in the tail domain after triggering TLR4, with several sites located in the TH2 domain and one threonine in the PH domain within the TH1 region [34]. Although these data indicate a regulatory mechanism in the action of these myosins in macrophage function against pathogens, no further evidence has been reported. The function of these two myosins seems to be redundant in contributing to lipopolysaccharide-triggered macrophage spreading [35]. In the context of macrophages as antigen presenting cells, MYO1E may control the exocytosis of cytoplasmic vesicles to the plasma membrane (containing major histocompatibility complex class II) through the interaction with the ARF7 effector protein (ARF7EP; also known as ARL14) and contributing to antigen presentation [36]. Consequently, the lack of MYO1E correlates with a deficient antigen-specific T cell proliferation [35].

More recently, it has been reported that MYO1F is induced in colonic macrophages and positively influences αVβ3-integrin accumulation [37]. This process enhances intercellular adhesion between macrophages and stimulates a proinflammatory (M1) phenotype by inducing integrin-linked kinase (ILK)/Protein Kinase B (AKT)/ (mammalian Target of Rapamycin (mTOR) signaling, which, in turn, induces Signal transducers and activators of transcription(STATS), STAT1 and STAT3 activation. Consequently, macrophages lacking MYO1F show reduced intercellular association via integrin-β3 and do not commit to the M1 phenotype. Furthermore, MYO1F upregulation leads to enhanced secretion and production of interleukin-1β and, accordingly, lack of MYO1F has been shown to result in reduced inflammation in a colitis model [37].

More recent data have shown that MYO1E and MYO1F are both required for efficient Fc receptors (FcR)-mediated phagocytosis [38]. Engagement of FcR on the phagocyte with antibodies on the target surface induces phagocytic cup formation (an actin-rich cup-like structure) to engulf the target. The plasma membrane is extended around the phagocytic cup and the ensuing closure of the cup results in phagosome formation where further processing of the target will occur. All these steps require active cytoskeleton dynamics and mechanical forces [31,39]. Macrophages form circular dynamic actin waves in the extending arms of the phagocytic cup, an event linked to phagocytosis [40]. MYO1E and F are reported as being recruited to the phagocytic cup, and they are found to be enriched in the punctate actin that makes up the wave. This location is dependent on the actin-binding via the motor domain, as well as the TH2 domain (via phospholipid membrane binding through the basic amino acids present in this domain), and thus it is exclusive to long-tailed unconventional class I myosins. The study of Barger et al. suggests a model where MYO1E and F function to tether the plasma membrane to the actin to successfully anchor the target to the cell, allowing actin polymerization within the cup to progress and finish internalization. The absence of MYO1E-F alters the local membrane tension and actin polymerization, resulting in an actin-dense phagocytic cup that leads to a slower closure of the phagocytic cup and lower rate of phagocytosis [38].

### 3.3. MYO1F in Mast Cells

Mast cells are essential effector cells in both the innate and adaptive arms of the immune system. They rely on their ability to migrate to inflammatory sites and release specific mediators stored in preformed granules or synthesized de novo if they are to function [41]. These processes are highly regulated by signaling events and precise cytoskeletal dynamics. Mast cells are resident in tissues, meaning that their migration as progenitors is needed before recruitment to inflammatory target tissue is possible [42]. Increased numbers of mast cells in inflamed tissue occur not only in bacterial and parasitic infection, but also in asthma and urticarial [43]. In these settings, mast cells recognize chemotactic stimuli and trigger signaling cascades that lead to integrin activation, adhesion, and migration. Stem cell factor (the tyrosine-protein kinase KIT receptor ligand) is the key chemotactic factor in mast cell proliferation, survival, homing, and migration [44].Recently, our group showed that MYO1F is expressed in mast cells, where it colocalizes with the cortical actin ring [21]. We also found that 3BP2 interacts with MYO1F. The cytoplasmic adaptor protein 3BP2 contains a PH domain, SH3-binding proline-rich regions, and a C-terminal SH2 domain [45], and it has roles in mast cell degranulation [46], survival [47], and migration [21]. Interestingly, the 3BP2–MYO1F interaction is modulated by KIT receptor signaling, possibly by the increase of phosphoinositide 3-kinase (PI3K) activity and consequently the production of phosphatidylinositol-3,4,5-triphosphate, ligands of the PH domains contained in 3BP2 and MYO1F, resulting in a major recruitment of both at the plasma membrane. 

Consequently, KIT inhibition alters MYO1F and 3BP2 translocation to the membrane, and subsequently colocalization of both molecules [21]. In the context of KIT signaling, the absence of MYO1F or 3BP2 impairs integrin-mediated mast cell adhesion and migration. MYO1F silencing specifically achieves this by decreasing the expression of two integrin β chains on the cell surface, β1 (Cluster of Differentiation 29) and β7, usually coupled with the α4 chain [21].

**Figure 2:**
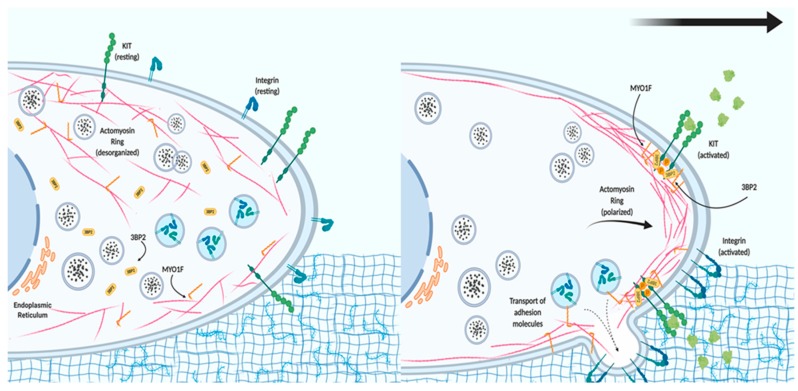
The role of MYO1F in mast cell migration. In resting conditions, mast cells express tyrosine-protein kinase KIT receptor and different integrins on the cell membrane. The actin cytoskeleton and MYO1F are distributed along the cell membrane and in the cytoplasm, whereas Src homology 3 domain (SH3)-binding protein 2 (3BP2) is mostly found in the cytoplasm. Activation of the KIT receptor initiates actin remodeling, which generates the leading edge (consisting of actin filaments) necessary for cell movement. MYO1F is necessary for the secretion and localization of activated integrin molecules that will induce adhesion to the extracellular matrix and aid cell migration.

These data point to a model where MYO1F could serve as a link between the actin cytoskeleton and the localization and function of integrin in the cell membrane after the activation of KIT receptors (Figure 2). We further hypothesize that MYO1F could modulate integrin by regulating the cortical actin mesh, on the basis of evidence that the cortical actin ring is necessary for mast cell migration [48]. Myo1E has not been reported in mast cells to date.

### 3.4. MYO1E in B Lymphocytes

The migration of lymphocytes to lymph nodes is a crucial step for the immune response to encounter antigens [49]. The adhesion of lymphocytes to high endothelial venules and their migration through that network are regulated by adhesins, integrins, and chemokines, as well as the actin cytoskeleton [50,51]. In lymphocytes, the expression of long-tailed unconventional class I myosins appears to be restricted to MYO1E. Its expression is especially high in B lymphocytes, but until recently, its role has been elusive. Consistent with its role in other immune cells, MYO1E has been reported as being critical for the recruitment and adhesion of activated B cells to the inguinal lymph node. Rolling and cellular transmigration are affected in activated B lymphocytes from MYO1E KO mice by a reduction of cell spreading (a mechanism used to maximize cellular contact to allow transmigration) due to a lack of CARMIL (capping protein, Arp2/3, and Myosin-I linker), which is important in cell migration and a ligand of MYO1E [52,53]. On the other hand, activated B cells from MYO1E KO mice have reduced levels of LFA-1 (Lymphocyte Function associated Antigen 1), CD44, and VLA-4 (Very Late Antigen-4) at the plasma membrane, suggesting that MYO1E is playing a role in vesicle trafficking of these adhesion molecules. The molecular mechanism involves the focal adhesion kinase (FAK), which has a role in integrin-mediated signal transduction [54]. FAK activity is reduced in activated B lymphocytes from MYO1E KO mice as well as AKT and the RAC-1 GTPase, both dependent on PI3K activity [53]. An explanation for the reduction in FAK activity in the MYO1E KO model may be by the fact that MYO1E interacts with FAK and promotes its autophosphorylation upon stimulation with CXCL12 in activated B cells [53]. This MYO1E–FAK interaction has been reported previously in melanoma, which we discuss in the next section. 

## 4. Class I Unconventional Long-Tailed Myosins in Diseases

Considering the role of long-tailed myosin, any improper function can lead to aberrant cell adhesion and migration, thereby compromising homeostasis and resulting in pathology. Altered function has been associated with various diseases.

### 4.1. MYO1E and MYO1F in Cancer

Several recent studies have focused on the roles of myosins in cell invasion and migration and on their potential role as tumor suppressors or activators in cancer [55].

#### 4.1.1. MYO1E

MYO1E directly interacts with FAK, a protein involved in many biological processes, including cancer [56]. This interaction, which occurs through a FERM-kinase linker domain, induces conformational changes and Y397 autophosphorylation that is essential for in vivo FAK activity. Once active, FAK accumulates in the nucleus, where it causes changes in gene expression (osteopontin and other fibronectin-type matrix genes) that are often seen in aggressive cancers. The increase of FAK activity and consequently of its target genes has been directly associated with metastatic events [57,58]. FAK and MYO1E interaction can be involved in the molecular basis of tumoral processes associated with FAK-dependent activity, such as melanoma. Indeed, the levels of phosphorylated nuclear phosphor Y379 FAK have been found to be high in patient-derived tissues and cell lines [56]. Moreover, both FAK and MYO1E have been found to promote mammary tumor progression [59,60,61].

The role of MYO1E in breast cancer has become more evident in recent years. An increase of MYO1E expression was found as a gene signature in patients with poor outcome in basal-like breast cancer (BLBC) [62]. This is a particularly aggressive molecular breast cancer subtype characterized by a specific gene cluster expressed by epithelial cells located in the basal or outer layer of the mammary gland. BLBC is a major clinical challenge because of its high relapse and prevalence in young women. More recently, a meta-analysis of human data has shown a correlation between high MYO1E expression and poor prognosis in BLBC and grade-1 breast cancer [61]. To delve into the role of MYO1E in breast cancer, studies have been performed with MYO1E knockout mice carrying the MMTV-PyMT transgene (mouse mammary tumor virus–polyoma middle T antigen). MMTV is a promoter that is expressed exclusively in mammary epithelium, thus conferring tissue specificity in this accepted mouse breast cancer model [63]. MYO1E KO have shown reduced cell proliferation and enhanced cell differentiation compared with MYO1E wild-type controls. The molecular mechanism underlying this effect could be related to the cell–cell junction dynamics. Although, the authors did not find differences in the localization of structural components of cell–cell junctions, the functional data provides evidence of reduced cell permeability in the MYO1E KO mice. Thus, the cell–cell junction in MYO1E WT is different compared with MYO1E KO, and may account for the distinct tumor morphology, proliferation, differentiation, and progression [61]. Further analysis will be needed in order to understand the molecular basis.

MYO1E is a component of the invadosome core that contributes to regulating their dynamics [64]. Invadosomes are actin-rich adhesion structures containing a dense meshwork of actin filaments at their center, as well as a ring of adhesion adapter proteins representing a site of intense matrix degradation by tumor cells. These play an important role in tumor cell invasion [65,66]. Targeting MYO1E activity may have therapeutic potential by preventing the metastasis of tumor cells that depend on invadosomes for migration. Furthermore, MYO1E expression may be used as a biomarker of highly invasive tumors.

#### 4.1.2. MYO1F

A recent study has shown that mutated MYO1F alters the mitochondrial network and induces tumor proliferation in thyroid cancer [67]. This arises due to a mutated MYO1F gene at the *TCO* locus (Gly134Ser) that leads to increased oncogenic potential in vitro in terms of cell growth and invasion. The mitochondrial membrane potential was similar in mutant versus control cells, suggesting that the mitochondria were still functional. However, in the mutant cell lines, analysis by live-cell visualization revealed that the mitochondria appeared as separate rod-shaped organelles with increased mass that produced significantly more intracellular and extracellular reactive oxygen species. How MYO1F regulates the mitochondrial network needs to be addressed.Several somatic mutations in MYO1F related to different types of cancer have been found in genetic databases [67]. One example is the fusing of the mixed lineage leukemia gene with the MYO1F gene, which has been described as recurrent in infant acute monocytic leukemia [68,69]. Interestingly, a VAV1-MYO1F fusion protein (where the SH3 domain of VAV1 is replaced by the SH3 domain of MYO1F) has been found in a heterogeneous non-Hodgkin peripheral T cell lymphoma. The aberrant fusion protein increases the oncogenic ability of VAV1 and is associated with a poor prognosis. What derives from these data is the fact that the MYO1F SH3 domain, possibly by recruitment of proteins, increases the VAV1-dependent tumor activity [70,71]. 

### 4.2. MYO1E in Kidney Diseases

MYO1E regulates actin reorganization during cell–cell adhesions in podocytes, major components of the glomerular filtration barrier. The podocyte actin cytoskeleton and its highly specialized cell–cell junctions (also called slit diaphragm complexes) play key roles in controlling glomerular filtration. MYO1E expression is enriched in these slit diaphragm complexes [72] and is recruited to the newly formed cell–cell junctions [73]. Moreover, MYO1E functions by linking the plasma membrane and actin filaments early in the process of cytoskeletal rearrangements in the newly stablished cell–cell contact in renal podocytes. To accomplish this, the TH1 and TH2 domains target MYO1E to the cell junctions, and the SH3 domain serves as an effector that interacts with the tight junction protein ZO-1 [74], therefore regulating the formation and dynamics of cell–cell junctions and compromising tissue permeability [73]. Consistent with this, MYO1E KO and podocyte-specific MYO1E KO show defects in podocyte organization and disruption of glomerular filtration, showing an increase in proteinuria, complement and IgG deposition. Interestingly, the observed renal defects do not seem to be immune mediated but rather result from impaired podocyte functions [75,76]. Furthermore, MYO1E mutations are associated with childhood-onset, glucocorticoid-resistant, focal segmental glomerulosclerosis that manifests as nephrotic syndrome with hypoalbuminemia, proteinuria, hyperlipidemia, and edema [77]. MYO1E may function as a linker between the plasma membrane phospholipids, protein components of cell–cell junctions, and actin filaments, maintaining the integrity of the glomerular filtration barrier for proper kidney function.

### 4.3. MYO1F in Hearing Loss

Myosins are necessary for hearing because they contribute both to the structure of stereocilia found in hair cells of the inner ear [78] and to auditory mechanotransduction. Specifically, MYO1F is expressed in the inner ear [15], and mutations in its gene may be responsible for nonsyndromic deafness [79,80]. Such mutations have been detected in the head domain, where they could affect ATPase activity or the actin binding process. However, it is not known whether hearing loss occurs due to an increased susceptibility to infection or to an abnormal neutrophil response, leading to the production of reactive oxygen species and the subsequent involvement of proteolytic enzymes and antimicrobial proteins.

## 5. Conclusions and Perspectives

Long-tailed unconventional class I myosins are involved in several physiological roles from endocytosis to cell migration, and these must be finely regulated to avoid pathology. The presence of specific domains, such as SH3 in their tail region, appears to be responsible for their individual cellular functions by facilitating association with adaptors and other binding proteins. In this way, the interaction between MYO1F and 3BP2 links the KIT receptor, which is involved in mast cell migration to the actin cytoskeleton. Alternatively, the interaction between MYO1E and FAK modulates the activity of the latter, and is important in cell proliferation, survival, and cellular motility. Both MYO1F and MYO1E regulate actin cytoskeleton dynamics and allow biological events such as phagocytosis for the clearance of pathogens, dead cells, and aberrant proteins, thereby promoting health. Cell migration is also critical for fighting infection and for allowing tumor cells to metastasize. Improving our understanding of the function and regulation of MYO1F and MYO1E could lead to developments that affect diseases, ranging from immune system alterations to cancer. Ultimately, it is hoped that we may identify specific myosin inhibitors or activators that have therapeutic potential.
